# Buprenorphine Facilitates Rapid Weaning From Very-High-Dose Intrathecal Hydromorphone

**DOI:** 10.7759/cureus.59134

**Published:** 2024-04-27

**Authors:** Thomas R Hickey, Ashok K Manepalli, James M Hitt

**Affiliations:** 1 Anesthesiology, Yale University School of Medicine, VA Connecticut Healthcare System, West Haven, USA; 2 Anesthesiology, Northeast Anesthesia and Pain Specialists, Concord, USA; 3 Anesthesiology, VA Western New York Healthcare System, Buffalo, USA

**Keywords:** buprenorphine, chronic pain management, opioid-induced hyperalgesia, opioid taper, intrathecal opioids

## Abstract

Pain management in patients on chronic opioid therapy is a common clinical challenge. The phenomena of opioid-induced hyperalgesia and tolerance are important contributors to that challenge. There are multiple strategies described to wean opioid doses and/or transition patients off opioids altogether. However, there is very little data to guide transitions off chronic intrathecal opioids. Here, we report on two patients with intractable post-laminectomy pain syndrome, resulting in severe functional limitation in the setting of opioid escalation culminating in the intrathecal delivery of hydromorphone to daily doses as high as 20 mg/day. We describe their rapid successful weaning off opioids using low-dose buprenorphine, which resulted in a dramatic improvement in pain and function.

## Introduction

Chronic opioid therapy for non-malignant pain was once generally accepted by the medical community without concerns over the use of high doses, but those views have been questioned in light of the lack of long-term outcome studies demonstrating benefits and mounting evidence of harms [1,2]. Despite the evolving view of chronic opioid therapy in this setting, approximately 22% of US adults with chronic pain use prescription opioids [3], and pain specialists are often involved in managing opioid medications that were initiated and titrated by other providers. Opioid-induced hyperalgesia (OIH) presents a unique challenge in this setting.

OIH has been examined in experimental settings, perioperative management [4], and opioid use disorder treatment [5], but less is known about it in the context of opioid therapy for chronic pain [6]. OIH results in a paradoxical increase in pain, suffering, hyperalgesia, and allodynia despite the escalation of opioid therapy. Prior case reports have documented OIH resulting from intrathecal fentanyl [7], morphine [8], and sufentanil [9]. Opioids, especially after chronic exposure, also create a state of tolerance, where escalating doses are required to achieve the same result.

Differentiating OIH from tolerance in chronic opioid therapy can be difficult as both mechanisms result in deteriorating pain control over time. While tolerance typically responds to increased opioid doses, OIH is expected to intensify with increasing doses [10]. Furthermore, pain practitioners are divided on the prevalence and optimal treatment of OIH stemming from chronic opioid therapy [11]. The two processes are also not mutually exclusive; patients in this situation may experience diminishing returns from increasing opioid doses without displaying signs of overt OIH (increased pain with increased opioid doses). Since patients on chronic opioids invariably develop dependence, attempts to wean opioids may result in increased pain secondary to withdrawal, further confusing the etiology of pain and treatment effects of chronic opioid therapy. This effect often results in discontinuation of the taper before expected benefits, including improved pain control, are realized.

Multiple treatment strategies have been used to address OIH in patients on chronic opioid therapy. A systematic review of published case reports of OIH encountered during chronic pain treatment described a variety of treatments, including ketamine, dexmedetomidine, lidocaine, and opioid weaning in a variety of malignant and non-malignant pain cases [12]. Some reports accomplished discontinuation of opioid therapy while others completed an opioid rotation with dose reduction, but none used buprenorphine to facilitate opioid discontinuation due to OIH. Meanwhile, large organizations, including the US Departments of Defense and Veterans Affairs, recommend consideration of buprenorphine in patients receiving daily opioids for pain [13].

Written consent was obtained from the patient described in Case 1 after he reviewed the case summary. Attempts to obtain written consent from the patient described in Case 2 were exhausted; therefore, protected health information was de-identified.

## Case presentation

Case 1

The patient was a 66-year-old male with a history of multiple lumbar laminectomies with instrumentation in the 1990s resulting in post-laminectomy pain syndrome treated with chronic opioid therapy. After failing oral opioid pharmacotherapy, he underwent intrathecal pump placement after a successful single intrathecal morphine trial. The catheter was documented at the T9 to T10 level. Intrathecal morphine monotherapy was initially used, and given a lack of benefit, treatment was transitioned to intrathecal hydromorphone monotherapy with a brisk titration, reaching a maximum dose of 20 mg/day after a few years without any benefit to his quality of life.

A dye study was performed by an outside physician with incomplete documentation; however, the patient experienced significant sedation and respiratory depression after injection of contrast that fully reversed with naloxone, consistent with an inadvertent bolus of intrathecal medication during the dye study and suggesting patency of the catheter system. The intrathecal hydromorphone was subsequently weaned to 16 mg/day with reports of constant 8-10/10 pain and functioning limited to only basic activities of daily living and self-care. After a few years of treatment at this dose, the patient transitioned care to one of the authors (JMH).

At this point, the patient complained of both stabbing and aching back pain and neuropathic pain radiating into the left lower extremity without a dermatomal pattern. A dye study was not repeated given complications reported from the previous attempt, but thoracic MR imaging was obtained to query catheter tip granuloma and showed no extrinsic compression of the thecal sac or spinal cord in the lower thoracic levels. Intrathecal bupivacaine was added, and the hydromorphone dose was gradually decreased to 7.5 mg/day over the course of two years. His pain was stable but severe (8/10) throughout the process. The patient felt further weaning would not be tolerable and optimizing intrathecal bupivacaine (10 mg/day) and clonidine (225 mcg/day) produced minimal benefit and did not facilitate further weaning of hydromorphone. He continued to report axial lumbar spinal pain with burning and shooting pain throughout the entire left lower extremity. As pain worsened, he suffered greater interference with daily activities, poor sleep, repeated falls due to vertigo, and worsening depression. The case was complicated by severe mixed central/obstructive sleep apnea that was untreated due to mask intolerance.

OIH had been suspected during the treatment, but options for further dose reductions were limited by severe pain. As the pump reached the end-of-life interval, the patient was presented with the option to discontinue intrathecal therapy with a transition to sublingual buprenorphine. Of note, no aberrancy was detected on urine toxicology or prescription refill intervals, and sublingual buprenorphine was chosen because of his extraordinary opioid dose. The transition was completed in the inpatient setting with continuous telemetry and respiratory monitoring. Prior to admission, he reported average pain of 8-9/10 with daily flares of 10/10. He scored 40/50 (80%) on the Oswestry Disability Index over four weeks leading to buprenorphine induction.

Saline was placed in the intrathecal reservoir; it took approximately 12 hours for the saline to flow through the internal and external pump tubing and reach the patient. Eighteen hours later, withdrawal symptoms were mostly mild, including rhinorrhea, loose stools, and nausea, which was controlled with two doses of sublingual buprenorphine 2 mg. His pain began to improve during the first day after discontinuation of intrathecal therapy and another dose of buprenorphine was not required for the next 24 hours. As such, he has transitioned to buccal buprenorphine 300 mcg and only required one dose the following day (48 hours post-saline infusion). Pain decreased to 3-5/10, and he was discharged home after 52 hours under observation with a prescription for 150 mcg of buccal buprenorphine, but he chose to discontinue buprenorphine secondary to the taste of the medication. He reported some mild nausea and diarrhea at home at 72 hours, which resolved within one day. His back pain remained mild to moderate, he elected to discontinue buprenorphine completely, and he was sent for pump explantation. In the succeeding months, he remained off opioids with improvement in pain, mood, mobility, breathing, sleep, and mental clarity. In addition, his Oswestry Disability Index improved from 80% to 56% (27 of 50 points). He did continue to report neuropathic left leg pain that partially responded to trials of duloxetine, pregabalin, and, later, oxcarbazepine, resulting in 4-6/10 pain.

Case 2

The patient in his mid-forties has a past surgical history significant for a multilevel lumbar decompression and fusion that occurred several years prior to the authors' involvement in his care (JMH). Due to inadequate benefit from oral pharmacotherapy (including high-dose oral opioids, gabapentinoids, duloxetine, and muscle relaxants) and interventional pain procedures, an intrathecal pump was implanted after a successful single-shot intrathecal morphine trial. The catheter was documented at the T9 to T10 level. He was initially treated with escalating doses of intrathecal morphine and was later transitioned to intrathecal hydromorphone after failed benefit from titration of intrathecal morphine. 

When the author took over management of his case (JMH), he was receiving opioid monotherapy with intrathecal hydromorphone 7.5 mg/day and 30 mg of oral oxycodone every four hours as needed, yet continued to suffer moderate pain with frequent severe exacerbations caused by movement and static posturing and limitations in function outside of activities of daily living. He reported mostly stabbing and aching pain in the lumbar spine without significant radicular or neuropathic components of pain. His functioning was limited to basic activities of daily living and self-care, and he was unable to engage in hobbies or gainful employment. Inasmuch as this had been a consistent pattern clinically on a stable opioid dose for many years, no imaging or dye studies were performed.

Subsequently, urine toxicology testing revealed non-prescribed opioids on two occasions within a few months of one another, which prompted counseling and a reduction of the intrathecal and oral regimen by 25%. He reported severe, unrelenting pain and intensifying functional disability. A third urine toxicology test revealed non-prescribed benzodiazepines, so the patient was informed that he would need to be transitioned off high-dose intrathecal therapy due to the risks of overdose. Given that substance use disorder may have caused his behaviors, referral to a methadone treatment program was offered, but he adamantly denied opioid use disorder and declined.

Due to concerns about overdose from non-prescribed opioids and benzodiazepines in the setting of high-dose intrathecal opioid therapy, an opioid taper was initiated. His oral oxycodone was stopped, saline was placed in the intrathecal pump reservoir, and the pump was set to deliver the lowest programable dose (0.7 mg/day); given the low pump infusion rate, it took approximately four days for the saline to flow through the internal and external pump tubing and reach the patient. Opioid withdrawal symptoms presented within 24 hours and sublingual buprenorphine 4 mg was initiated. Over the first two days, the dose was adjusted to 4 mg every six hours (16 mg daily), which resulted in a reduction of his static 10/10 pain to the moderate range (5-7/10) without any significant withdrawal symptoms or adverse effects.

Eventually, his average pain dropped to 4/10 with gradual improvement in functional ability, including engaging in hobbies and seeking employment. The benefit he perceived after the transition to sublingual buprenorphine caused him to reevaluate his reliance on opioids as analgesics. He reported no cravings or desires to return to full agonist opioid therapy, and he eventually requested an opioid taper with the goal of discontinuing all opioid therapy. The buprenorphine was slowly weaned to 2 mg/day over the succeeding eight months, and he transitioned to transdermal buprenorphine in the final two months to complete the discontinuation of opioids. The patient’s pain and function remained significantly improved from baseline without further aberrancy. He required no further medical or interventional pain treatments and was sent for pump explantation.

## Discussion

The cases presented illustrate the challenges of pain management in patients on high-dose chronic opioid therapy, and the high doses of intrathecal hydromorphone encountered are well beyond doses typically encountered in patients on any opioid formulation of opioid pharmacotherapy. Reports of the oral to intrathecal conversion ratio range widely from 12:1 to 90:1 to 300:1 [14-16]. A survey of pain experts querying intravenous to intrathecal found 100:1 to be the most common opinion, correlating to a 300:1 oral to intrathecal conversion [17]. Thus, as a rough estimate, if 7.5 mg intrathecal hydromorphone is approximately equivalent to 50 mg intrathecal morphine, the commonly accepted 300:1 conversion equates to 15,000 daily oral morphine. Whatever conversion is accepted, these patients were on daily oral morphine equivalents orders of magnitude above what is commonly prescribed.

The patients felt that they were benefiting from this therapy despite ongoing severe pain and disability. Weaning attempts were limited by worsening pain and functional disability. Buprenorphine was chosen to facilitate opioid rotation because of its ability to treat both pain and opioid withdrawal symptoms, and the patients tolerated cessation of intrathecal therapy with only mild withdrawal. They also reported striking and rapid improvements in pain and function during the buprenorphine induction.

Buprenorphine is a partial agonist opioid with a low to intermediate µ receptor efficacy [18]. It has a high affinity for the µ receptor [19], is highly lipophilic [20], and has a long duration of analgesia [21]. Its mechanism of analgesia is thought to be primarily via the µ receptor agonism [22], although agonism at the opioid receptor-like-1 (nociceptin-orphanin FQ opioid peptide receptor) may contribute [23]. Partial agonism, together with the minimal activation of β-arrestin recruitment [24,25], likely results in several advantages compared to other opioids in pain management: a ceiling effect for respiratory depression [26], reduced euphorigenic effects, reduced gastrointestinal adverse effects [27], and reduced OIH in experimental and clinical studies [28,29]. In animals, sustained morphine exposure results in neurotransmitter changes, including increases in the endogenous κ-agonist dynorphin, and induces plasticity in both primary afferents and spinal cord neurons, contributing to OIH [30]. Buprenorphine is a κ receptor antagonist that competes with the pronociceptive actions of dynorphin [30]. As such, buprenorphine is uniquely capable of facilitating rapid opioid tapering and treatment of OIH [31].

OIH has been studied in the context of surgery, but less is known about the development of OIH in the setting of chronic opioid therapy [32]. A large systematic review of published studies of non-surgical OIH found that the detection of OIH depended on the means of assessment, and none of the studies identified increased pain from baseline levels [33]. The presence of OIH must be suspected in patients on chronic opioid therapy, particularly at high doses, when pain and functional disability remain high despite ongoing treatment, but the definitive diagnosis of OIH remains elusive.

These cases demonstrate that buprenorphine is effective in rapidly weaning patients with presumed OIH off very high daily intrathecal opioid doses and that buprenorphine may treat both opioid withdrawal and OIH. The presented patients were successfully weaned from daily oral morphine equivalents in the thousands of milligrams via initial low-dose buprenorphine titrated to a maximum daily dose of 16 mg, which facilitated the rapid discontinuation of opioid therapy. This resulted in rapid improvements in pain, function, and quality of life, suggesting that OIH was likely playing a significant role in their persistent pain states. It also provides clinical support in the chronic pain population for the advantageous neuroadaptations (µ receptor re-sensitization and upregulation) observed in buprenorphine inductions for opioid use disorder [34].

While there is little published on buprenorphine-mediated transitions from intrathecal opioids, a similar positive result was obtained in a 61-year-old female patient receiving 6.4 mg/day intrathecal hydromorphone. In that case, the intrathecal hydromorphone dose was gradually reduced as sublingual buprenorphine was increased from 0.5 mg once daily to a final discharge dose of 4 mg twice daily on day 8, at which time the intrathecal hydromorphone was at minimal flow rate [35].

## Conclusions

Chronic opioids, especially at high doses, create unique challenges for pain management. This may be especially true in the case of chronic intrathecal administration, where there is little published data to guide management, and little still describing management with buprenorphine. Meanwhile, large healthcare systems are recommending the consideration of buprenorphine in patients on chronic opioid therapy. Here, we describe the successful rapid transition from high-dose intrathecal therapy by leveraging the unique properties of buprenorphine, which resulted in the discontinuation of opioid therapy and dramatic improvements in pain and function.
